# Magnesium supplementation and high volume hydration reduce the renal toxicity caused by cisplatin-based chemotherapy in patients with lung cancer: a toxicity study

**DOI:** 10.1186/2050-6511-15-70

**Published:** 2014-12-04

**Authors:** Takako Oka, Tatsuo Kimura, Tomohiro Suzumura, Naoki Yoshimoto, Toshiyuki Nakai, Norio Yamamoto, Kuniomi Matsuura, Shigeki Mitsuoka, Naruo Yoshimura, Shinzoh Kudoh, Kazuto Hirata

**Affiliations:** Department of Respiratory Medicine, Graduate school of Medicine, Osaka City University, 1-4-3 Asahi-machi, Abeno-ku, Osaka, 545-8585 Japan; Department of Premier Preventive Medicine, Graduate school of Medicine, Osaka City University, 1-1-43 Abenosuji, Abeno-ku, Osaka, 545-6090 Japan; Department of Medical Oncology, Izumi Municipal Hospital, 4-10-10 Fuchu-cho, Izumi, Osaka, 594-0071 Japan; Department of Clinical Oncology, Graduate school of Medicine, Osaka City University, 1-4-3 Asahi-machi, Abeno-ku, Osaka, 545-8585 Japan

**Keywords:** Magnesium, Low volume hydration, Cisplatin, Lung cancer

## Abstract

**Background:**

Renal toxicity is a clinical problem that affects 28 − 42% of patients undergoing treatment with cisplatin. Renal toxicity can be minimized by high volume hydration with mannitol diuresis. Recent reports have shown that cisplatin induces depletion of Mg and that Mg supplementation can reduce renal toxicity. We hypothesized that Mg infusion combined with low volume hydration may not be sufficient to overcome cisplatin-induced renal toxicity.

**Methods:**

In total, 85 patients with lung cancer receiving their first cycle of cisplatin-based chemotherapy at the Osaka City University Hospital were classified into three groups: those administered high volume hydration without Mg infusion (high-volume Mg-), high volume hydration with Mg infusion (high-volume Mg+), and with low volume hydration with Mg infusion (low-volume Mg+). Serum creatinine (sCr) and creatinine clearance (CrCl) were examined before and after treatment with cisplatin. Multivariable analysis was carried out to identify the most important contributing factors.

**Results:**

There were no significant differences in pre-treatment sCr levels or CrCl between groups. In the high-volume Mg- group, post-treatment sCr significantly increased compared with pre-treatment levels, while post-CrCl significantly decreased compared with pre-treatment CrCl (p < 0.001 and p < 0.001, respectively). In the high-volume Mg+ group, there was no significant difference between pre- and post-treatment levels of sCr, or between pre- and post-treatment CrCl (p = 0.118 and p = 0.254, respectively). In the low-volume Mg+ group, there was a trend towards increased sCr levels and decreased CrCl after treatment (p = 0.068 and p = 0.055, respectively). Multivariate analysis revealed that the absence of Mg infusion and low-volume hydration were both independent factors for decreased CrCl (p < 0.001 and p = 0.001, respectively).

**Conclusions:**

High-volume hydration and Mg infusion reduces the renal toxicity induced by cisplatin. A low-volume Mg+ regimen may be considered for patients with adequate renal function. Trial Registration: Observational Study UMIN000013950; Registered 13 May 2014

**Electronic supplementary material:**

The online version of this article (doi:10.1186/2050-6511-15-70) contains supplementary material, which is available to authorized users.

## Background

The mortality rate associated with lung cancer is increasing worldwide. In fact, lung cancer (along with tracheal and bronchial cancers) was responsible for 1.5 million (2.7%) deaths in 2011, compared with 1.2 million (2.2%) deaths in 2000, according to the World Health Organization. In more than 60% of patients with lung cancer, particularly those with non-small cell lung cancer (NSCLC), the cancer is inoperable at the time of diagnosis and requires chemotherapy [1].

Cisplatin is one of the most important components of standard therapies for lung cancer and is also an effective adjuvant for chemotherapy and chemoradiotherapy [2]. Renal toxicity is a well-known adverse effect of cisplatin treatment, which limits its use. Despite intensive prophylactic measures, renal toxicity still affects a large proportion of patients treated with cisplatin, and a significant decrease in glomerular filtration rate is commonly observed during treatment [3]. In 1977, Cvitkovic *et al.* reported that cisplatin-induced renal toxicity could be avoided with substantial hydration and mannitol-induced diuresis [4]. Subsequently, clinical trials were initiated in several types of cancers, including epidermoid carcinoma, ovarian adenocarcinoma, germ cell tumors of the testis, and metastatic melanoma [5, 6]. Hayes *et al*. reported about patients who had been pre-hydrated intravenously with 2,000 ml of 5% glucose in one-half normal saline over 12 h, and post-hydrated with 1,200 ml of one-half normal saline over 6 h. Mannitol was infused (50 ml of 25% mannitol) as an intravenous bolus just before the administration of cisplatin, and was continuously infused (300 ml of 20% mannitol) for 6 h after the administration of cisplatin [6]. Even if cisplatin was approved in 1984 for use in Europe and the United States, nephrotoxic damage still affects 28 − 42% of patients treated with cisplatin [7].

To date, a number of studies have investigated strategies for prevention of cisplatin-induced renal toxicity. As free cisplatin causes renal toxicity, shortening the free cisplatin and renal tubular contact time is important to reduce its nephrotoxicity [8]. As more than 90% of free cisplatin binds to plasma proteins within 4 h, hydration within that time after initiating treatment is a critical factor in reducing toxicity [8]. In addition, previous reports have demonstrated that cisplatin-induced Mg depletion may occur in some patients [9]. Willox *et al.* revealed that supplementation with 16 mEq Mg was beneficial in reducing renal tubular damage in patients with testicular cancer receiving cisplatin [10], while Bodnar *et al.* demonstrated that 40 mEq Mg supplementation had nephroprotective effects during chemotherapy with cisplatin in patients with epithelial ovarian cancer [7]. Muraki *et al.* showed that hydration with 8 mEq Mg and mannitol without furosemide prevents the nephrotoxicity induced by cisplatin and pemetrexed in patients with advanced NSCLC [11]. Hirai, *et al.* also showed that 20 mEq Mg supplementation may be beneficial in preventing cisplatin-induced nephrotoxicity in patients with esophageal or hypopharyngeal cancer [12]. Yoshida *et al*. reported that 8 mEq Mg preloading before cisplatin administration significantly reduced cisplatin-induced nephrotoxicity in 496 patients with thoracic malignancies [13]. Therefore, Mg supplementation, in addition to hydration, may also reduce the renal toxicity induced by cisplatin. Therefore, Mg supplementation is being considered an option for cisplatin-based chemotherapy. Prospective studies since 2007 have examined the use of low-volume hydration in combination with Mg supplementation. Tiseo, *et al.* demonstrated that low-volume hydration with 16 mEq Mg supplementation in cisplatin-based chemotherapy led to no increase in creatinine in only 35.3% of patients [14]. Hotta *et al*. reported that low-volume hydration (2,500 ml) with 4 mEq Mg supplementation before and after cisplatin administration was associated with slightly worse renal toxicity during all cycles of chemotherapy, without significance [15]. Horinouchi *et al.* examined the safety of low-volume hydration (1,550 − 2,050 ml) with 8 mEq Mg supplementation; however, renal function during all courses of cisplatin administration was slightly worse [2]. However, few studies have compared the renal toxicities associated with treatment using high-volume hydration with Mg supplementation to those associated with treatment using low-volume hydration with Mg supplementation. We hypothesized that Mg infusion may not be sufficient to overcome the renal toxicity caused by cisplatin during low-volume hydration. Therefore, we performed a historical prospective cohort study wherein patients were classified into three groups: high-volume hydration without Mg infusion, high-volume hydration with Mg infusion, and low-volume hydration with Mg infusion.

## Methods

### Patients

This study was reported according to the STROBE guidelines [16]. The chemotherapy procedure was explained and informed consent was obtained from all patients before treatment. The Mg/hydration protocols were approved by the Institutional Review Board (No. 1402) of Osaka City University Hospital in accordance with the Declaration of Helsinki.

The renal toxicity in patients with lung cancer receiving their first cycle of cisplatin-based chemotherapy at the Osaka City University Hospital between December 2011 and October 2013 were included in the present study. Patients were placed into three groups, and renal toxicity was compared before and after cisplatin-based chemotherapy. In our hospital, until December 2012, we performed conventional high-volume hydration without Mg infusion in patients undergoing cisplatin-based chemotherapy (high-volume Mg-). In December 2012, we initiated the administration of Mg sulfate during the pre-hydration stage (high-volume Mg+). Subsequently, the new low-volume hydration with Mg infusion regimen was initiated in July 2013 (low-volume Mg+). We investigated patient characteristics including age, sex, body surface area, performance status, stage, histology, serum hemoglobin, serum albumin, serum creatinine (sCr), creatinine clearance (CrCl), cisplatin dose, regular use of non-steroid anti-inflammatory medicines, regular use of Mg-containing medications for purgative, and combined anticancer agents.

### Treatments

All patients received combination chemotherapy of cisplatin and another cytotoxic agent (i.e. vinorelbine, pemetrexed, irinotecan, etoposide, gemcitabine, S-1, amrubicin, or docetaxel). Table 1 shows an example of the chemotherapy hydration regimen in each group. In the high-volume Mg- and high-volume Mg+ groups, patients were administered a total of 3,550 ml of electrolyte-containing solutions that contained normal saline and one-quarter saline solution Soldem 3A (Terumo, Tokyo, Japan) on day 1 and 950 ml of the solutions on days 2 and 3. A total of 600 ml of 20% mannitol and furosemide (20 mg) was administered as enforced diuresis on day 1, and 300 ml of 20% mannitol was administered on days 2 and 3. Antiemetic agents (dexamethasone [6.6 mg] and granisetron [3 mg]) were infused on days 1–3. Cisplatin, which was dissolved in 300 ml of normal saline solution, was infused over 90 min. In the low-volume Mg+ group, patients were administered a total of 2,200 ml of electrolyte-containing solutions on day 1 with no infusions on days 2 and 3; instead, they were administered 2,000 ml of water per os on day 1 and 1,000 ml of water on days 2 and 3. A total of 300 ml of 20% mannitol was infused before cisplatin administration on day 1. Antiemetic agents (fosaprepitant meglumine [150 mg], dexamethasone [6.6 mg], and palonosetron [0.75 mg]) were administered on day 1. Cisplatin, which was dissolved in 100 ml of normal saline solution, was administered over 45 min. In the high- and low-volume Mg+ groups, Mg sulfate was administered before cisplatin in one-quarter saline solution. The dose of Mg sulfate was 8 mEq based on previous reports [2, 11]. Blood chemistry was analyzed twice a week during treatment. The chemotherapy procedure was explained and informed consent was obtained from all patients. Since this was a historical prospective cohort study, the difference from the previous Mg- protocol was explained to patients in high volume Mg+ group, and the patients in low volume Mg+ group were informed about the difference from the previous high volume Mg+ protocol.

**Table 1:** **An example of the chemotherapy hydration regimen for each group**

		High-volume hydration Mg-		High-volume hydration Mg+		Low-volume hydration Mg+	
			ml		ml		ml
Day 1		N.S.	50	N.S.	50	N.S.	50
	Antiemetics					N.S.	100
		Granisetoron 3 mg	100	Granisetoron 3 mg	100	Fosaprepitant meglumine 150 mg	
		Dexamethasone 6.6 mg	2	Dexamethasone 6.6 mg	2	N.S.	50
						Palonosetoron 0.75 mg	5
						Dexamethasone 9.9 mg	2
	Other cytotoxic agent	N.S. with other cytotoxic agent	500	N.S. with other cytotoxic agent	500	N.S. with other cytotoxic agent	100
	Pre-hydration	N.S.	500	N.S.	500	1/4 saline solution	500
		1/4 saline solution	500	1/4 saline solution	500	MgSO_4_ 8 mEq	
				MgSO_4_ 8 mEq			
	Diuresis			20% Mannitol	300	20% Mannitol	300
	Cisplatin	N.S. with cisplatin	300	N.S. with cisplatin	300	N.S. with cisplatin	100
	Post-hydration	N.S.	500	N.S.	500	N.S.	500
	Diuresis	20% Mannitol	300				
	Post-hydration	1/4 saline solution	500	1/4 saline solution	500	1/4 saline solution	500
	Diuresis	20% Mannitol	300	20% Mannitol	300		
		Frosemide 20 mg	2	Frosemide 20 mg	2		
	Oral hydration		0		0		2,000
Day 2,3		N.S.	50	N.S.	50		
	Antiemetics	Granisetoron 3 mg	100	Granisetoron 3 mg	100		
		Dexamethasone 6.6 mg	2	Dexamethasone 6.6 mg	2		
	Hydration	1/4 saline solution	500	1/4 saline solution	500		
	Diuresis	20% Mannitol	300	20% Mannitol	300		
	Oral hydration		0		0		1,000
	Total infusion	Day 1	3,550	Day 1	3,550	Day 1	2,200
		Day 2,3	950	Day 2,3	950	Day 2,3	0

N.S., normal saline.

### Renal function and gastrointestinal toxicity

Changes in sCr and gastrointestinal toxicity/anorexia were assessed using Common Terminology Criteria for Adverse Events Version 4.0 (CTCAE v4.0). sCr was measured just before chemotherapy (pre-sCr), twice a week during treatment, and on the last day of the first cycle of chemotherapy (post-sCr). CrCl was calculated using the Cockcroft-Gault formula: CrCl [ml/min] = (140 – age [years] × weight [kg]) × 0.85 [if female]/(72 × sCr [mg/dl]). ΔsCr was calculated using the formula: ΔsCr [mg/dl] = (sCr [mg/dl] after chemotherapy) – (sCr [mg/dl] before chemotherapy). ΔCrCl was calculated using the formula: ΔCrCl [ml/min] = (CrCl [ml/min] before chemotherapy) – (CrCl [ml/min] after chemotherapy).

### Statistical analysis

Chi-square and Fisher’s exact tests were used for categorical comparisons of data. Differences in the means of continuous measurements were tested with a Student’s t-test and the Mann-Whitney U test. The paired t-test and Wilcoxon matched-pairs signed-rank test was used for paired continuous measurements. The influence of the following factors on changes in CrCl were investigated on univariate analysis: age, sex, performance status, serum albumin, cisplatin dose, regular use of non-steroid anti-inflammatory medicines, regular use of Mg-containing purgatives, ≥grade 2 anorexia, Mg infusion, and low-volume hydration. All of the factors were then included in a multiple linear regression model to identify contributing factors. P values < 0.05 were considered statistically significant. All statistical analyses were performed with the statistical package Stata 12 for Windows.

## Results

### Patient characteristics

A total of 89 patients were enrolled. Four patients whose complications or medical histories may have affected their renal function were excluded; these included cases of a carcinoma of unknown primary, nephrotic syndrome, renal cancer, and a patient who received an inadequate amount of Mg sulfate. Eighty-one patients had lung cancer, while the remaining had thymic and bronchial cancers. Forty-one patients were included in the high-volume Mg- group, 27 in the high-volume Mg+ group, and 17 in the low-volume Mg+ group. Table 2 shows the characteristics of the 85 eligible patients. There were no significant differences in clinical and pathological parameters between the three groups. We analyzed the number of chemotherapy cycles and major reasons for treatment discontinuation (Additional file 1: Table S1). In the high-volume Mg- group, the most common event leading to discontinuation was renal toxicity (n = 9, [22%]), followed by generalized weakness (n = 5, [12%]). On the other hand, in the high- and low-volume Mg+ groups, it was seen that none and 1 patient, respectively, discontinued cisplatin treatment due to renal toxicity. The reasons for discontinuation in these groups were other adverse reactions, switching to another regimen, and cancer progression.

**Table 2:** **Patient characteristics**

	High volume hydration Mg-	High volume hydration Mg+	Low volume hydration Mg+
	(n = 41)	(n = 27)	(n = 17)
Age (year)	66 (63 to 70)	66 (57 to 70)	63 (62 to 67)
Gender Male (%)	31 (75.6)	18 (66.6)	16 (94.1)
BSA (m^2^)	1.7 ± 0.2	1.6 ± 0.2	1.7 ± 0.2
PS (ECOG): 0 (%)	5 (12.1)	4 (14.8)	5 (29.4)
1 (%)	36 (87.8)	23 (85.2)	12 (70.6)
Stage **I** (%)	0 (0)	1 (3.7)	0 (0)
**II** (%)	5 (12.2)	3 (11.1)	3 (17.6)
**III** (%)	25 (61.0)	15 (55.5)	8 (47.1)
**IV** (%)	9 (22.0)	7 (25.9)	6 (35.3)
Others (%)	2 (4.9)	1 (3.7)	0 (0)
Histology Adenocarcinoma (%)	17 (41.5)	13 (48.1)	11 (64.7)
Squamous cell carcinoma (%)	11 (26.8)	7 (25.9)	5 (29.4)
Small cell carcinoma (%)	9 (22.0)	6 (22.2)	1 (5.9)
Others (%)	4 (9.8)	1 (3.7)	0 (0)
Serum Hb (g/dl)	13.3 (12.0 to 14.8)	13.6 (12.8 to 14.8)	13.3 (12.7 to 14.9)
Serum Alb (g/dl)	4.0 ± 0.4	4.1 ± 0.4	3.9 ± 0.4
Cisplatin dose (mg/m^2^)	80 (80 to 80)	80 (75 to 80)	80 (75 to 80)
Regular use drug			
NSAIDs (%)	10 (24.4)	2 (7.4)	2 (11.7)
Mg for purgative (%)	14 (34.1)	8 (29.6)	5 (29.4)
Combined anticancer agent (%)			
VNB	22 (53.7)	13 (48.1)	10 (58.8)
PEM	3 (7.3)	6 (22.2)	5 (29.4)
CPT-11	3 (7.4)	1 (3.7)	0
VP-16	8 (19.5)	6 (22.2)	1 (5.9)
GEM	2 (4.9)	0	1 (5.9)
S-1	1 (2.4)	1 (3.7)	0
AMR	1 (2.4)	0	0
DTX	1 (2.4)	0	0

Continuous variables are expressed as mean ± standard deviation or median (interquartile range). Categorical variables are expressed as number (proportion).

BSA, body surface area; PS, performance status; Hb, hemogulobin; Alb, albumin; NSAIDs, non-steroid anti-Inflammatory drugs; Non-Steroidal Anti-Inflammatory Drugs; Mg for purgative, Mg-contating medications for purgative; VNB, vinorelbine; PEM, pemetrexed; CPT-11, irinotecan; VP-16, etoposide; GEM, gemcitabine; AMR, amurubicin; DTX, docetaxel.

### Renal toxicity

**Figure 1:**
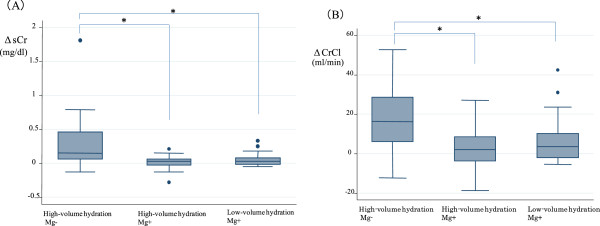
**Comparisons of ΔsCr (A) and ΔCrCl (B) between treatment groups.** The box plot provides information about the median, variability, and outliers of data distribution. The horizontal line within each box indicates the sample median. The plot consists of a box that extends from the 25th quartile to the 75th quartile. The box lines that extend from each end to the outermost data point that falls within the distances were computed as follows: 1st quartile +1.5 (interquartile range) and 3rd quartile +1.5 (interquartile range). Data points outside these computed ranges were considered outliers. sCr, serum creatinine; CrCl, creatinine clearance, *P < 0.05.

Table 3 shows the maximum grade of sCr increase in each group. In the high-volume Mg- group, the grade was significantly higher than that in the high- and low-volume Mg+ groups (p < 0.001 and p = 0.02, respectively). Grade 1 renal toxicity was seen in 96.3% and 88.2% of patients in the high- and low-volume Mg+ groups, respectively. Next, we examined the recovery of sCr and CrCl after cisplatin treatment. Table 4 shows the comparison between pre- and post-treatment values of sCr and CrCl in each group. No significant differences in sCr or CrCl were present between the three groups before treatment. In the high-volume Mg- group, post-sCr significantly increased compared with pre-sCr, while post-CrCl significantly decreased compared to pre-CrCl (p < 0.001 and p < 0.001, respectively). In the high-volume Mg+ group, there was no significant difference between pre- and post-sCr levels or between pre- and post-CrCl (p = 0.118 and p = 0.254, respectively). In the low-volume Mg+ group, there was a trend towards an increase in post-sCr compared with pre-sCr levels, and a trend towards a decrease in post-CrCl compared to pre-CrCl (p = 0.068 and p = 0.055, respectively). Figure 1 shows the comparison of ΔsCr and ΔCrCl between treatment groups. The ΔsCr in the high-volume Mg- group (median, 0.15 mg/dl; interquartile range, 0.06 to 0.46 mg/dl) was significantly higher compared with ΔsCr in the high-volume Mg+ (median, 0.03 mg/dl; interquartile range, −0.03 to 0.06 mg/dl) and low-volume Mg+ groups (median, 0.03 mg/dl; interquartile range, −0.02 to 0.08 mg/dl) (p < 0.001 and p = 0.0035, respectively). The ΔCrCl in the high-volume Mg- group (median, 16.29 ml/min; interquartile range, 5.96 to 28.68 ml/min) was also significantly higher than ΔCrCl in the high-volume Mg+ (median, 1.99 ml/min; interquartile range, −3.79 to 8.56 ml/min) and low-volume Mg+ groups (median, 3.63 ml/min; interquartile range −2.09 to 10.13 ml/min) (p < 0.001 and p = 0.0316, respectively). We also analyzed the sCr of each group at the end of subsequent cycles. The median values of sCr at the end of therapy in the high-volume Mg- and low-volume Mg+ groups were significantly increased compared with pre-sCr using the paired t-test and Wilcoxon matched-pairs signed-rank test (p < 0.001, and p = 0.01, respectively) (Additional file 2: Table S2).

**Table 3:** **The maximum grade of sCr increased and anorexia in each group**

Grade (n/%)	High-volume hydration Mg- (n = 41)	High-volume hydration Mg+ (n = 27)	Low-volume hydration Mg+ (n = 17)	***P***value (High Mg- vs High Mg+)	***P***value (High Mg- vs Low Mg+)
	**sCr**				
0	0 (0)	1 (3.7)	1 (5.9)	<0.001	0.002
1	22 (53.6)	26 (96.3)	15 (88.2)		
2	17 (41.5)	0 (0)	1 (5.9)		
3	2 (4.9)	0 (0)	0 (0)		
	**anorexia**				
< 2	33 (80.4)	21 (77.8)	14 (82.4)	0.787	0.592
≥ 2	8 (19.5)	6 (22.2)	3 (17.6)		

The maximum grade of sCr increase was assessed using the Common Terminology Criteria for Adverse Events Version 4.0 (CTCAE v4.0).

**Table 4:** **The comparison between pre and post sCr, and CrCl in each group**

	Pre sCr	Post sCr	***P***value	Pre CrCl	Post CrCl	***P***value
	(mg/dl)	(mg/dl)		(ml/min)	(ml/min)	
**High-volume hydration Mg-**	0.75	0.92	<0.001	78.88	65.99	<0.001
	(0.64 to 0.92)	(0.79 to 1.21)		(70.37 to 95.64)	(48.62 to 80.13)	
**High-volume hydration Mg+**	0.71	0.71	0.118	73.21	73.66	0.254
	(0.58 to 0.84)	(0.63 to 0.84)		(67.93 to 104.61)	(64.39 to 100.65)	
**Low-volume hydration Mg+**	0.72	0.73	0.068	83.59	76.99	0.055
	(0.68 to 0.78)	(0.70 to 0.85)		(72.51 to103.63)	(72.22 to 85.69)	

Median (interquartile range). sCr, serum creatinine; CrCl, creatinine clearance.

### Univariate and multivariate analyses

Table 5 shows the results of univariate and multivariate analyses. Only Mg infusion was a good predictor in univariate analysis (p < 0.001). In multivariate analysis, Mg infusion was a significant good predictor of ΔCrCl and low-volume hydration was a significant poor predictor of ΔCrCl (p < 0.001 and p = 0.001, respectively).

**Table 5:** **The predictive factors of ΔCrCl according to univariate and multivariate analyses**

	Univariate analysis		Maltivariate	
	Coefficient [95% CI]	***P***value	Coefficient [95% CI]	***P***value
Age (per 5 years)	−1.15 [−3.74 to 1.43]	0.375	−1.58 [−3.74 to 0.58]	0.149
Female (vs male)	5.03 [−2.40 to 12.46]	0.182	5.63 [−1.64 to 12.91]	0.127
PS 1 (vs PS 0)	9.16 [−1.37 to 19.68]	0.087	3.34 [−4.82 to 11.49]	0.418
Serum albumin	−3.53 [−11.89 to 4.84]	0.404	−1.28 [−9.14 to 6.59]	0.747
Cisplatin dose (per 5 mg/ml)	−1.41 [−1.57 to 4.38]	0.348	1.24 [−1.40 to 3.90]	0.35
Regular use drug				
NSAIDs	7.63 [−1.75 to 17.02]	0.109	2.98 [−5.25 to 11.21]	0.473
Mg for purgative	0.54 [−7.25 to 8.34]	0.89	−3.67 [−10.10 to 2.76]	0.259
Anorexia (grade 2 or more)	−2.13 [−9.51 to 5.24]	0.566	2.19 [−5.31 to 9.70]	0.562
Mg infusion	−14.73 [−21.25 to −8.21]	<0.001	−15.28 [−21.99 to −8.57]	<0.001
Low volume hydration	−3.40 [−11.33 to 4.53]	0.396	22.31 [9.16 to 35.46]	0.001

CI, confidential interval; PS, performance status; NSAIDs, non-steroid anti-Inflammatory drugs; Mg for purgative, Magnesium containing medications for purgative.

## Discussion

We found that renal function was significantly worse after cisplatin treatment in the high-volume Mg- group, and it also tended to worsen in the low-volume Mg+ group. Only the high-volume Mg+ group showed no significant alterations in renal function after cisplatin treatment. Multivariate analysis indicated that both the absence of Mg infusion and low-volume hydration were independent predictors for decreased renal function.

Mg supplementation and high-volume hydration may protect against the renal toxicity induced by cisplatin [7, 10, 11]. Although the detailed mechanism underlying this effect is not clear, recent reports have shown that the human organic cation transporter 2 (OCT2) is predominantly expressed in the human kidney at the basolateral membrane of renal proximal tubules [17]. In mice, Oct2 has been implicated in the cellular uptake of cisplatin, and *Oct1/Oct2*-deficient mice are protected from severe cisplatin-induced renal tubular damage [17]. In rats, Mg depletion may cause dehydration and up-regulation of rat Oct2, which enhances renal accumulation of cisplatin and subsequent acute kidney injury [18]. In humans, a single nucleotide polymorphism in the *OCT2* gene *SLC22A2* (rs316019) has been associated with reduced cisplatin-induced nephrotoxicity and decreased gene expression [17]. In renal proximal tubule cells, the accumulation of cisplatin from the basolateral side is significantly greater than that from the apical side [19]. Thus, Mg supplementation was subsequently proposed to prevent Mg depletion and renal toxicity.

The volume of fluid for hydration is also an important factor that affects cisplatin-induced renal toxicity [4, 20, 21]. To our knowledge, no previous reports have compared renal toxicity between patients receiving high-volume Mg+ and low-volume Mg+ regimens. In the present multivariate analysis, both the volume of hydration and Mg infusion contributed to increased renal protection. These protocols were based on previous reports. Recent prospective studies have investigated low-volume hydration with Mg supplementation using hydration volumes of 1,500 − 2,500 ml [2, 14, 15]. In our study, the volumes of pre-hydration fluid administered were 1,800 ml in the high- and 900 ml in the low-volume Mg+ groups. However, the volumes of post-hydration fluid administered within 4 h were almost similar between the high- and low-volume Mg+ regimens. The difference in volume may thus be an important factor related to the observed differences in renal toxicity. Hydration volume is related to osmolality and both serum and urinary concentrations of cisplatin. The extent of cisplatin induced nephrotoxic injury is dependent on both dose and cumulative cisplatin levels. Since high-volume hydration reduces the concentration of cisplatin compared to low-volume hydration, low plasma levels of cisplatin should reduce renal accumulation and decrease nephrotoxicity. In addition, high volume hydration decreases osmolality of blood resulting in decreased secretion of antidiuretic hormone, decreased water reabsorption, more diluted urine, and increased urine output. It can thus be hypothesized that low urinary levels of cisplatin reduce renal accumulation of cisplatin and decrease nephrotoxicity. Therefore, the differences in osmolality in these two protocols may be related to the observed differences in nephrotoxicity.

One remaining question is whether Mg supplementation will also reduce the cytotoxic effects of cisplatin on tumor cells. Laboratory and clinical findings of Mg depletion have been observed in patients treated with high-dose cisplatin. Under low Mg conditions, tumor suppressors such as p21 and p27 are up-regulated, while oncogenes such as cyclins D and E are down-regulated [22]. It has also been suggested that there may be a correlation between the availability of Mg and vascular endothelial growth factor [23], and in particular that Mg depletion may suppress tumor cell proliferation and neoangiogenesis. Therefore, there could be some concern that Mg supplementation might promote tumor cell proliferation. However, previous prospective clinical trials and retrospective studies have indicated that Mg supplementation does not affect tumor response to cisplatin-based chemotherapy [7, 10, 11]. At any rate, the relationship between Mg depletion and tumor response to chemotherapy will require further investigation.

The present study has some limitations. First, this was a historical prospective cohort study and included a relatively small number of patients. Second, while some patients had consumed 2,000 ml of water on day 1 and 1,000 ml of water on days 2 and 3, the optimal amount of fluid intake was not evaluated. Indeed, many previous examinations of low-volume hydration involved oral hydration on days 2 and 3 rather than infusion; however, the effect of oral hydration has not yet been examined. Third, no data on the levels of serum Mg levels was available. It is known that serum hypomagnesemia reflects Mg depletion, but serum Mg is only 1% of the total Mg present in the body [24]. In some cases, levels of serum Mg may not be decreased even in the presence of Mg depletion. Cisplatin treatment is one such case, and Mg supplementation is needed even in the absence of serum hypomagnesemia. This renders it difficult to know the optimal amount of Mg supplementation. Nonetheless, our study revealed that even 8 mEq of Mg sulfate reduced renal toxicity. Fourth, renal function in cisplatin chemotherapy was assessed only after the first or last cycle. The renal damage by cisplatin may be cumulative. The knowledge of sCr in the following cycles would be important. In our study, in the high-volume Mg- group, the median value of sCr after the last cycle was significantly higher than after the first cycle (p = 0.004) (data not shown). This result suggests that the renal damage by cisplatin was cumulative with every cycle in Mg- group. Thus, Mg supplementation with high-volume hydration may have a much greater impact on renal damage caused by cisplatin.

## Conclusion

High-volume hydration and Mg supplementation can reduce the renal toxicity induced by cisplatin. Our data also suggest that a low-volume Mg+ regimen can be considered for patients with adequate renal function. Further prospective clinical studies in a larger patient population, including association with total body Mg levels and optimization of Mg intake, along with correlations between Mg depletion and tumor response to chemotherapy should be performed.

## Electronic supplementary material

Additional file 1: **Cisplatin chemotherapy administered.** (DOC 48 KB)

Additional file 2: **The comparison between pre and end of sCr in each group.** (DOC 42 KB)

## Abbreviations

high-volume Mg-: High volume hydration without Mg infusion
high-volume Mg+: High volume hydration with Mg infusion
low-volume Mg+: Low volume hydration with Mg infusion
sCr: Serum creatinine
CrCl: Creatinine clearance
NSCLC: Non-small cell lung cancer
CTCAE v4.0: Common Terminology Criteria for Adverse Events Version 4.0
Oct2: Organic cation transporter 2.
