# Supplementary material for: Magnesium supplementation and high volume hydration reduce the renal toxicity caused by cisplatin-based chemotherapy in patients with lung cancer: a toxicity study
Source: BMC Pharmacol Toxicol. 2014 Dec 4;15:70. doi: 10.1186/2050-6511-15-70 (PMC4272804; doi:10.1186/2050-6511-15-70)
Supplement: Supplementary file 1 [file 40360_2014_352_MOESM1_ESM.doc]

Additional file 1: Table S1				
Cisplatin chemotherapy administered				
	the number of chemotherapy cycles			
	1	2	3	4
high-volume hydration Mg	41	28	25	23
high-volume hydration Mg	27	23	18	17
low-volume hydration Mg-	17	14	12	12
