# Supplementary material for: Magnesium supplementation and high volume hydration reduce the renal toxicity caused by cisplatin-based chemotherapy in patients with lung cancer: a toxicity study
Source: BMC Pharmacol Toxicol. 2014 Dec 4;15:70. doi: 10.1186/2050-6511-15-70 (PMC4272804; doi:10.1186/2050-6511-15-70)
Supplement: Supplementary file 2 [file 40360_2014_352_MOESM2_ESM.doc]

Additional file 2: Table S2

The comparison between pre and end of sCr in each group.

	pre sCr (mg/dl)	end of sCr (mg/dl)	*P* value
**high-volume hydration Mg-**	0.75 (0.64 to 0.92)	1.05 (0.87 to 1.27)	<0.001
**high-volume hydration Mg+**	0.71 (0.58 to 0.84)	0.75 (0.66 to 0.86)	0.08
**low-volume hydration Mg+**	0.72 (0.68 to 0.78)	0.81 (0.72 to 0.89)	0.01

median (interquartile range). sCr, serum creatinine;
